# Structural Study Reveals That Disabling a Kinase Can Increase Sensory and Signaling Fidelity

**DOI:** 10.1371/journal.pbio.1001980

**Published:** 2014-10-28

**Authors:** Richard Robinson

**Affiliations:** Freelance Science Writer, Sherborn, Massachusetts, United States of America

**Figure 1:**
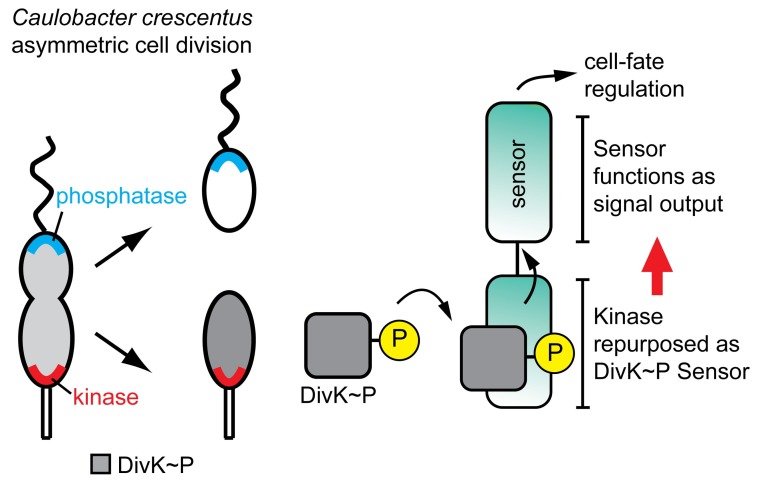
The bacterium *C. crescentus* divides asymmetrically to produce daughter cells with distinct cell fates. The authors reveal how an unusual pseudokinase (DivL) detects and processes a cell fate signal (DivK).

A fundamental strategy of asymmetric cell division is to place different signaling molecules in each nascent daughter cell, which then go on to drive the two cells toward different fates. In *Caulobacter crescentus*, a bacterial model used to study this process, a critical molecular driver is a protein called DivK. The phosphorylated form of DivK (DivK^∼^P) maintains the daughter cell that contains it in a stalked, division-competent, stem cell–like form, while the nonphosphorylated form drives the differentiation of the other daughter into a flagellum-powered “swarmer” cell, free to roam its environment in search of new food sources (Figure 1).

DivK interacts with another protein, DivL, whose presence is necessary for both turning on the gene program leading to swarmer cell differentiation and, conversely, for inhibiting that program in the stalked cell, but the molecular details of the interaction of the two proteins have been unclear. In this issue of *PLOS Biology*, W. Seth Childers, Qingping Xu, Ashley Deacon, Lucy Shapiro, and colleagues elucidate those details to show that DivL has taken an evolutionary path that allows it to serve as an unusual and highly specific sensor for the phosphorylation state of DivK.

The authors began by showing that DivL binds strongly and specifically to DivK^∼^P, but not to DivK. To explore the reasons for that specificity, they analyzed the crystal structure of DivL's so-called HK region. HK stands for histidine kinase, and DivL shares an evolutionary history and many structural features with other histidine kinases. One thing it does not share, though, is the eponymic histidine at the active core of the protein, having replaced it instead with a tyrosine, which, unlike the histidine, does not become phosphorylated as part of its normal action cycle. The significance of that replacement became clear when a histidine was substituted back in—cells survived, but the localization of DivL was altered, as was cell morphology and doubling time.

Like other HK's, DivL is a dimer, and the crystal structure revealed that the tyrosine sits near the interface between the two monomers. While the amino acid sequences of the monomers were identical, their conformation was not. Each monomer was made of several rigid substructures, whose orientations differed between the two monomers. In one monomer, two substructures, the small “input helix” and a larger substructure including the (nonfunctional) catalytic domain and the “output helix,” were tilted away from the axis of a third substructure, the two-helix “docking module” that connected to the tyrosine in the “input helix.” In the second monomer, these two structures were more aligned with the axis of the docking module. Mutation of several critical residues at the putative DivK^∼^P docking site did not prevent dimer formation but did significantly reduce DivK^∼^P binding, supporting the role of this site in linking the two proteins.

DivL also contains four PAS domains, a common signal-sensing structure found in both prokaryotic and eukaryotic organisms. To determine their effect on the DivL-DivK^∼^P interaction, the authors constructed DivL mutants with three, two, one, or no PAS domains. While the three-domain and two-domain mutants retained high specificity for the phosphorylated form of DivK, that specificity was lost entirely in the absence of any PAS domains, indicating that allosteric interactions among PAS domains and the other portions of the protein likely govern the selectivity of DivL for DivK^∼^P.

Canonical HK's are enzymatically active, but the authors showed that DivL was not, having neither detectable kinase nor phosphatase activity. That loss of activity, they suggest, is critical in allowing DivL to act primarily as a sensory protein, faithfully detecting the phosphorylation state of DivK without altering it through its interaction. A consequence of that loss of activity, they note, is that signal flow through DivL is the reverse of that through a typical histidine kinase. Instead of receiving a signal that then triggers phosphorylation of its substrate, DivL receives a signal regarding the phosphorylation state of its “substrate” DivK^∼^P and triggers downstream activity ultimately determining the fate of the daughter cell in which it resides (Figure 1).

The results in this study strengthen the understanding of asymmetric cell development in this important model organism. Whether a similar disabling of catalytic activity and subsequent “reverse flow” of information will be found in other cellular systems remains an open and intriguing question.

**Childers WS, Xu Q, Mann TH, Mathews II, Blair JA, et al. (2014) Cell Fate Regulation Governed by a Repurposed Bacterial Histidine Kinase.** doi:10.1371/journal.pbio.1001979

